# Congenital aortic stenosis due to unicuspid unicommissural aortic valve: a case report

**DOI:** 10.1186/s13019-018-0755-0

**Published:** 2018-06-07

**Authors:** Arnar B. Ingason, Gunnlaugur Sigfusson, Bjarni Torfason

**Affiliations:** 1 Department of Medicine, University of Iceland, Vatnsmyrarvegur 16, 101 Reykjavik, Reykjavik, Iceland; 2 Children’s Hospital, Landspitali University Hospital, Reykjavik, Iceland; 3 Department of Cardiothoracic Surgery, Landspitali University Hospital, Reykjavik, Iceland

**Keywords:** Unicuspid aortic valve, Congenital aortic stenosis, Aortic valve replacement, Case report

## Abstract

**Background:**

Unicuspid unicommissural aortic valve is an extremely rare congenital anomaly that usually presents in adulthood but can rarely present in infancy. We report a 17-year-old patient with congenital aortic stenosis secondary to unicuspid unicommissural aortic valve that was successfully treated with aortic valve replacement.

**Case presentation:**

The patient was diagnosed with aortic stenosis after a murmur was heard in the newborn nursery and subsequently underwent aortic balloon valvuloplasty 6 weeks after birth. He had been regularly followed up since and underwent numerous cardiac catheterizations, including another aortic balloon valvuloplasty at age 13. During follow-up at age 17, the patient presented with symptomatic severe aortic stenosis and mild left ventricular hypertrophy. Aortic valve replacement was planned since the patient was nearly adult-sized and to reduce the risk of cardiac decompensation. During the operation an unicuspid unicommissural aortic valve was revealed. The patient recovered well post-operatively. He was discharged 5 days after the surgery in good condition and was completely symptom-free at follow-up 6 weeks later.

**Conclusions:**

Unicuspid aortic valve is a rare congenital anomaly that can cause congenital aortic stenosis. It is seldom diagnosed pre-operatively but should be suspected in infants presenting with aortic stenosis.

## Background

Unicuspid aortic valve (UAV) is an extremely rare congenital malformation with an estimated prevalence of 0.02% in adults [1]. UAVs share many characteristics with bicuspid valves, such as premature valvular calcifications, aortic root dilations, and aortic dissection [2]. In unicuspid valves, these changes occur even more rapidly. UAVs most often present with aortic stenosis, either isolated or with concomitant aortic regurgitation [2, 3].

UAVs are divided into two subtypes; unicommissural and acommissural UAVs. Since acommissural UAVs have smaller aortic orifice compared to unicommissural valves, they have a more aggressive presentation and are usually symptomatic at birth [3]. Unicommissural UAVs generally present in the 4th to 6th decade of life [2, 4], but can rarely present at infancy [5]. We report a case of 17-year-old male with congenital aortic stenosis secondary to a unicommissural UAV that was successfully treated with aortic valve replacement (AVR).

## Case report

A 17-year-old male with congenital aortic stenosis presented to his pediatric cardiologist for follow-up. He had been diagnosed with aortic stenosis after a murmur was heard in the newborn nursery, and subsequently underwent aortic balloon valvuloplasty 6 weeks after birth. He had been regularly followed up since and underwent numerous cardiac catheterizations, including another aortic balloon valvuloplasty at age 13.

**Fig. 1:**
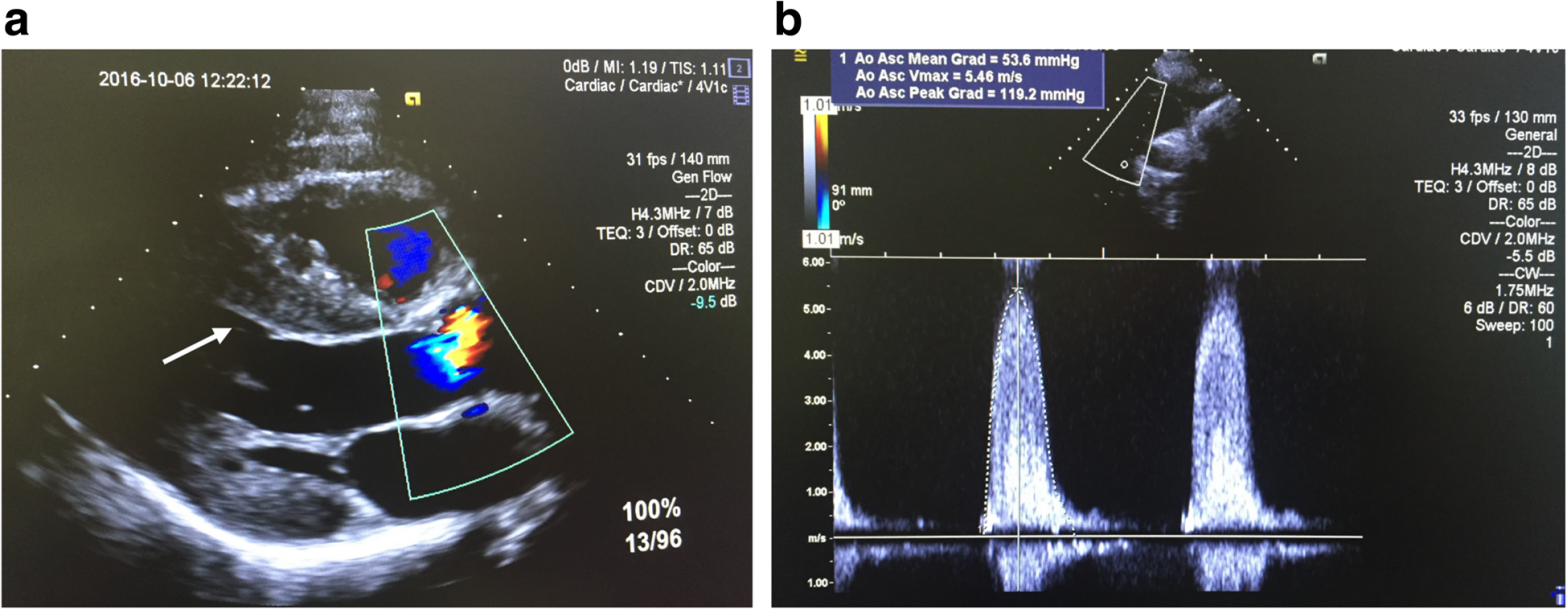
**a** Echocardiography demonstrating an increased septal diameter (arrow). **b** Echocardiography measurements demonstrating a mean gradient of 54 mmHg, maximal gradient of 119 mmHg, and peak flow of 5.5 m/s through the aortic valve orifice

**Fig. 2:**
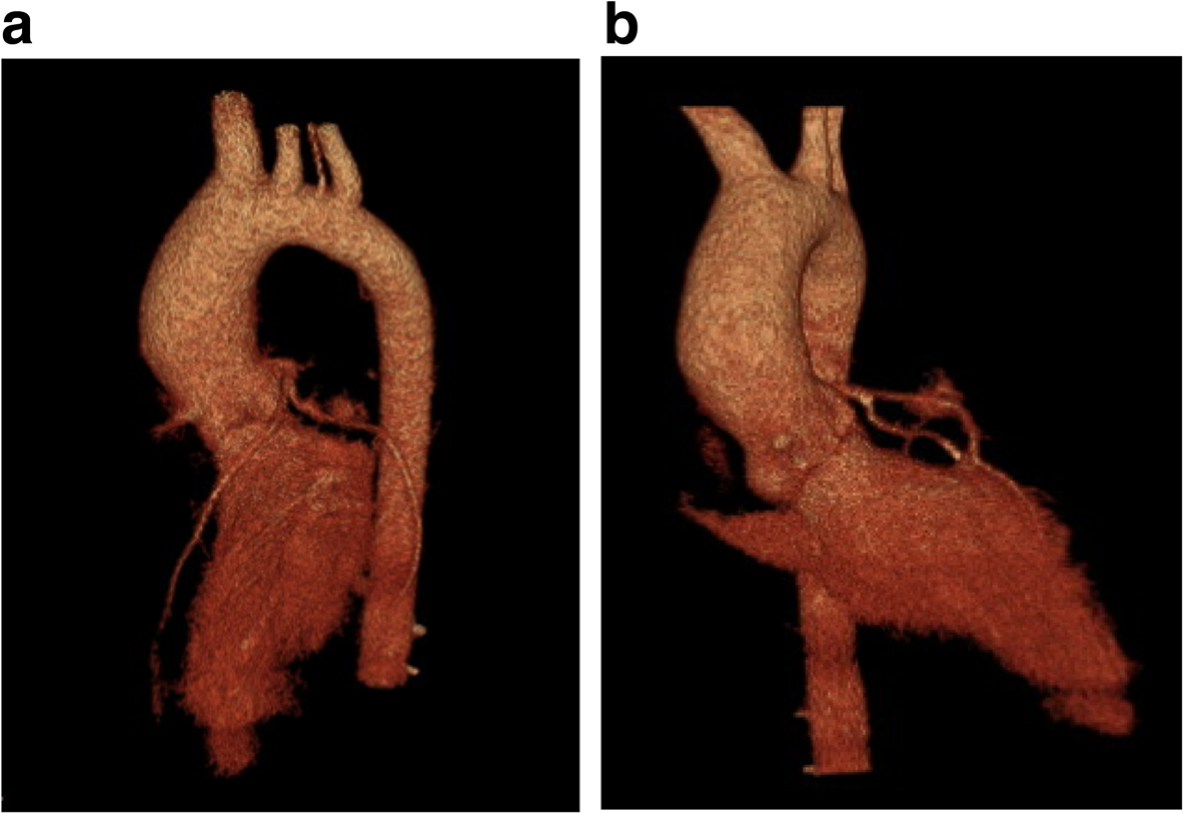
**a** Lateral and **b** anterior projection of a three-dimensional reconstructed CT angiography demonstrating a slightly dilated ascending aorta and normal sized aortic annulus

Upon presentation, echocardiography was performed and revealed a mean gradient of 54 mmHg, maximum gradient of 119 mmHg through the aortic valve orifice, aortic valve area of 0.4 cm^2^/m^2^, and septal diameter of 1.6 cm^2^ (Fig. 1). Subsequently, the patient was scheduled for AVR 3 weeks later. A CT angiography was obtained before surgery and revealed a mild ascending aortic dilation of 34.4 × 42.2 mm in maximal diameter, without increased annular size (Fig. 2).

During pre-operative examination, the patient admitted having dyspnea during exertion but had never experienced angina, palpations, or syncope. He reported being very physically active. A 4/6 crescendo/decrescendo systolic murmur was auscultated over the whole precordium, with the murmur radiating to the neck. Lung auscultation was clear and jugular venous distension was absent. His chest X-ray was normal with cardiac index within referral range. The patient and his parents expressed their desire for biologic valve implantation.

**Fig. 3:**
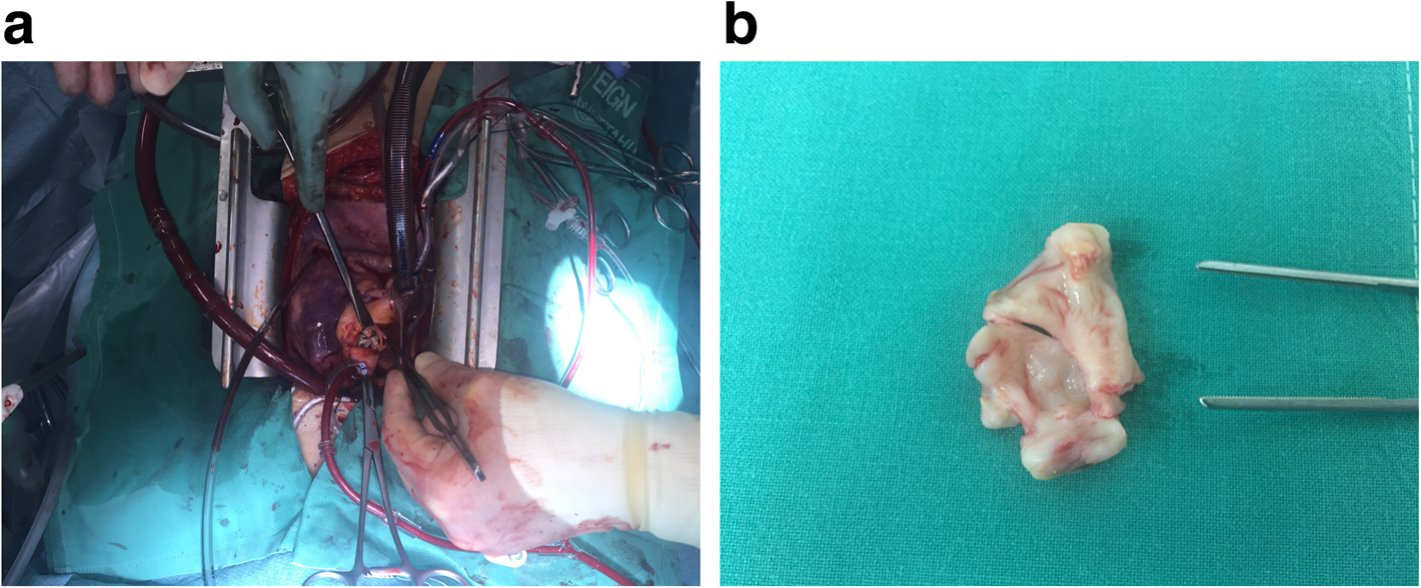
**a** The aortic valve revealed during surgery. **b** Macroscopic view of the resected unicommissural unicuspid aortic valve

AVR was performed under normothermic cardiopulmonary bypass. Following aortotomy an unicuspid unicommissural aortic valve was revealed, with a single commissure located just right of the left coronary ostium. The valve was thickened and extremely stenotic with mild calcification underneath the right coronary ostium (Fig. 3). The valve was removed in one piece using scissors and knife, and replaced with a 27 mm biologic Freestyle valve (Medtronic Inc., Minneapolis, Minnesota) using continuous 4–0 Prolene sutures. The stentless valve was implanted subcoronally to allow for a nonobstructive position for the right and left coronary ostia between the commissures of the newly implanted bioprosthesis. Finally, the ascending aorta was closed using continuous 4–0 Prolene sutures.

Post-operatively, there were no adverse events and the patient recovered well. He was discharged 5 days after surgery in good condition. At follow up 6 weeks later, the patient had returned to his daily activities and was completely symptom-free. An echocardiography revealed a functional valve with no regurgitation and insignificant gradient across the valve. The left ventricular hypertrophy was receding with a measured septal diameter of 1.2–1.4 mm. Left ventricular function was considered normal and no pericardial effusion was noted.

## Discussion and conclusions

UAV is a rare cause of aortic stenosis. Unicommissural UAVs are usually asymptomatic early in life, although they can rarely present at infancy, as in the previously described case. In a case series of 21 patients with unicommissural UAV, only 2 patients presented during the first year of life [5]. Even though the patient was diagnosed with aortic stenosis shortly after birth, the diagnosis of UAV was not made until open heart surgery was performed 17 years later. This is common, indeed the majority of UAVs are diagnosed peri-operatively [2, 6]. Transesophageal echocardiography (TEE) is the gold standard for diagnosis of UAV with a sensitivity and specificity of 75 and 86% respectively [7]. With evolving imaging technology, such as three-dimensional TEE, a higher frequency of pre-operative diagnosis is anticipated.

Congenital aortic stenosis due to UAV is most often treated with balloon valvuloplasty, surgical valvotomy, or commissurotomy [3]. AVR is generally not recommended until the patient has reached full size. This is due to higher mortality rates compared to adults as well as higher frequency of re-operation due to patient-prosthesis mismatch and structural valve degeneration [8]. If AVR is needed in infancy or early childhood, a Ross surgery is often recommended [9, 10]. In a Ross procedure, the aortic valve is replaced with the patient’s own pulmonary valve. The autograft has some capacity to grow along with the patient’s heart thereby reducing the risk of patient-prosthesis mismatch in a growing child. The main problem with the Ross procedure is that a simultaneous pulmonary valve replacement is required, thus converting a single valve disease into a double valve pathology [11]. The Ross procedure is more technically difficult than other valve replacement alternatives, with relatively high mortality, but has been shown to be safe in experienced hands [8].

When the patient presented pre-operatively, he had symptomatic severe aortic stenosis and mild left ventricular hypertrophy. AVR was planned since the patient was nearly adult-sized and to reduce the risk of cardiac decompensation due to further cardiac hypertrophy. In preparation for the AVR, the patient and his guardians expressed their desire for bioprosthetic valve replacement. Although mechanical valves have generally been recommended for patients younger than 60 years old, multiple factors have led to increased use of bioprosthetic valves in younger populations, including high re-operative survival rate, lifestyle expectations, and recent advances in transcatheter valve replacements. AHA/ACC guidelines for managing valvular heart diseases state that the choice of prosthetic valve type should be a shared decision and bioprosthetic valves should be recommended if anticoagulation is not desired [12]. Lifelong anticoagulation following mechanical valve replacement can be debilitating for physically active individuals and reduce quality of life. Additionally, the annual risk of major bleeding is about 3% for oral anticoagulants [13, 14] but has been reported as high as 4.4% following mechanical valve replacement [15]. The benefit of lower re-operative rates in mechanical valve replacements must be carefully weighed to the risk of bleeding complications and reduction in quality of life on a case-by-case basis.

In conclusion, UAV is a rare cause of aortic stenosis, but should be suspected in infants presenting with aortic stenosis. Although rarely diagnosed pre-operatively, TEE has relatively high sensitivity and specificity for the condition. AVR is generally not recommended until patients have reached full-size due to higher mortality and re-operation rates compared to adults. Instead, balloon valvuloplasty, surgical valvotomy, or commissurotomy are the initial treatments of choice.

## Abbreviations

AVR: Aortic valve replacement
CT: Computer Tomography
TEE: Transesophageal echocardiography
UAV: Unicuspid aortic valve
